# Oral Anticoagulant Discontinuation and Its Predictors in Patients with Atrial Fibrillation

**DOI:** 10.3390/jcm11206022

**Published:** 2022-10-12

**Authors:** Adane Teshome Kefale, Woldesellassie M. Bezabhe, Gregory M. Peterson

**Affiliations:** School of Pharmacy and Pharmacology, University of Tasmania, Private Bag 26, Hobart, TAS 7001, Australia

**Keywords:** atrial fibrillation, oral anticoagulant, discontinuation, persistence, Australia

## Abstract

Background: Oral anticoagulants (OACs) are important in reducing the risk of ischaemic stroke in people with atrial fibrillation (AF). Although patients need to take their OAC continuously, it has been suggested that discontinuation is common in clinical practice, and this could predispose patients to thrombotic complications. Aims: To investigate the rate of OAC discontinuation and its predictors in patients with AF, using national data from Australian general practices. Methods: We analysed data obtained from NPS MedicineWise’s MedicineInsight dataset. We included patients with a recorded diagnosis of AF who newly started an OAC between 1 January 2013 and 31 December 2017. Patients were considered persistent if an OAC was prescribed continuously without discontinuing more than 60 days gap in therapy. The follow-up period was 12 months post-initiation. Multivariable models were used for the analysis of predictors. Results: Of 16,075 patients included in the cohort, 47.3% were females, and the mean age was 74.6 (SD 10.2) years. The overall OAC discontinuation rate was 13.2% (confidence interval (CI) 12.6–13.7%) by 12 months post-initiation. The discontinuation rates for warfarin, apixaban, dabigatran and rivaroxaban were 18.3% (95% CI 17.2–19.5%), 10.1% (95% CI 9.2–11.0%), 10.9% (95% CI 9.4–12.5%) and 12.2% (95% CI 11.4–13.2%), respectively. Warfarin had a significantly higher risk of discontinuation compared to direct-acting OACs. Factors that are known to increase the risk of stroke (older age, diabetes, and hypertension) were associated with better persistence. Conclusions: A relatively high proportion of patients with AF continued OAC therapy by 12 months post-initiation. Positively, patients with the highest risk of stroke and lowest risk of bleeds seemed to have better persistence.

## 1. Introduction

Atrial fibrillation (AF) is the most common sustained arrhythmia and is associated with substantial morbidity, mortality, and healthcare expenditure [1,2]. In 2019, an estimated 60 million people were living with AF, and the age-adjusted prevalence was 744 per 100,000 people [3]. Australia is one of the high AF burden countries, and over half a million Australians had AF in 2019 [4].

AF increases the risk of stroke by almost five-fold, with 20–30% of all strokes being due to AF. It also increases the risk of death by 1.5-to 2-fold [5]. Oral anticoagulation reduces AF-associated strokes and mortality [6,7]. Compared to a placebo, warfarin reduced the risk of stroke by up to 70% in clinical trials [6]. Direct-acting oral anticoagulants (DOACs) showed non-inferior efficacy and better safety, relative to warfarin, for stroke prevention in people with AF. A meta-analysis of pivotal DOAC trials reported decreased stroke or systemic embolic events and all-cause mortality by 19% and 10%, respectively, compared to warfarin [7]. As a class, DOACs are associated with a reduced risk of intracranial haemorrhage [7,8] and increased risk of gastrointestinal bleeding [7,9], and a comparable rate of major bleeding to warfarin [7].

Studies conducted using Australian general practice data showed a substantial increase in the initiation of OAC therapy for stroke prevention in patients with AF over the past decade [10,11]. Once an OAC is initiated, the next crucial issue is the continuity of therapy. The clinical benefits of OAC therapy largely depend on its persistent use (usually life-long, unless contraindications arise). However, there is international evidence that persistence with OACs in clinical practice is often poor and progressively declines with time on therapy [12]. Research investigating persistence with OACs in patients with AF is limited in Australia. Studies by Simons et al. [13,14] used Pharmaceutical Benefits Scheme (PBS) data to assess OAC persistence for the period ending September 2016. However, the data source provided limited clinical information for predictor analysis and did not allow for identifying the specific indication for OAC use, particularly for warfarin. Therefore, this study aimed to assess the discontinuation rate of OACs post-initiation in patients with AF and identify associated risk factors using comprehensive data collected from general practices across Australia.

## 2. Methods

### 2.1. Data Source

The study was conducted using the NPS MedicineWise’s dataset, MedicineInsight. The dataset consists of de-identified longitudinal data from electronic medical records collected by consenting general practices, including patient demographics, diagnoses, pathology tests, prescribed medications, and clinical observations [15]. The dataset is widely used for research and is representative of the Australian population by age and sex [15,16,17]. As of September 2021, MedicineInsight had enrolled 680 general practices (8% of the national total) and included over 3.2 million regular patients [15]. The dataset has been previously described in detail [15,16].

### 2.2. Study Population and Follow-Up

We included adults (aged ≥ 18 years) with a documented diagnosis of AF who were newly commenced on an OAC between 1 January 2013 and 31 December 2017. Patients were considered new users if there was no evidence of OAC use in the 12 months before the index date (the date of the first OAC prescription identified during the study period) [18]. In addition, patients must have attended the same general practice at least three times in two consecutive years (baseline period and index year) [19]. Patients were excluded if they had a recorded OAC prescription before AF diagnosis or had a recorded diagnosis of venous thromboembolism within six months before the index date in the absence of a documented diagnosis of AF. Patient records were followed from the index date until discontinuation of the index OAC, last clinical encounter or after 365 days of follow-up, whichever came first.

### 2.3. Study Outcomes

The outcome of the study was the discontinuation of OAC therapy. We considered OAC therapy as being discontinued if the patient did not receive a new prescription of an OAC within 60 days after the end of the prior prescription coverage. On the Australian PBS, the number of days covered by a prescription with repeats varies with the type of OAC, ranging between 150 and 180 days. Since assessing warfarin’s days of coverage is challenging due to a high probability of subsequent dose adjustments depending on the international normalised ratio (INR) levels, 150 days of supply was considered with the assumption that a patient is taking one tablet per day at a stable dose [20].

Both persistence with the index OAC and switching between OACs was considered. Patients who switched to another OAC were considered persistent with OAC therapy. When a patient received a prescription before the exhaustion of the previous prescription, the supply for the next prescription was adjusted for the leftover from the previous coverage. If the patient switched to another OAC, any leftover from the index OAC was discarded and discontinuation was assessed based on the prescription coverage of the second OAC for the days after switching. The proportion of patients who remained on OAC therapy was calculated at 6 and 12 months post-initiation. Discontinuation rates were measured as cumulative incidences. Sensitivity analysis was conducted by changing the discontinuation gap to 30 and 90 days.

### 2.4. Study Covariates

Age was calculated on the index date, while clinical information was collected for the baseline period. The most recent haemoglobin and estimated glomerular filtration rate (eGFR) values recorded on or before the index date were used.

The general practice postcode provided by the Australian Statistical Geography Standard (ASGS) of the Australian Bureau of Statistics (ABS) was used to identify rurality. The ASGS classifies areas into five rurality categories based on relative access to services measured using the Accessibility and Remoteness Index of Australia (ARIA+). The ARIA+ score estimates the road distance of a point to the nearest urban centres and localities. The five categories are major cities (ARIA+ 0–0.20), inner regional (ARIA+ 0.21–2.40), outer regional (ARIA+ 2.41–5.92), remote (ARIA+ 5.93–10.53) and very remote (ARIA+ > 10.53) [21]. The last two were collapsed into one group for this analysis. The socioeconomic status of participants was estimated using the ABS Socio-Economic Indexes for Areas (SEIFA) decile based on the general practice’s postcode [22]. The SEIFA ranks areas from 1 (the most disadvantaged) to 10 (the most advantaged). We grouped areas into five SEIFA quintiles derived from deciles.

Comorbidities were identified from the condition flags provided by MedicineInsight [15]. Details of terms (both coded and non-coded) used to identify comorbidities are available in Table S1 and other publications that used the dataset [23]. The risk of stroke was calculated using CHA_2_DS_2_-VASc (Congestive heart failure (1 point), Hypertension (1 point), Age ≥ 75 years (2 points), Diabetes mellitus (1 point), Stroke/transient ischaemic attack (2 points), Vascular disease (1 point), Age 65–74 years (1 point) and Sex category (female, 1 point)) [24]. The risk of bleeding was estimated using the Outcomes Registry for Better Informed Treatment (ORBIT) score (Older age ≥ 75 years (1 point), Reduced haemoglobin (2 points), Bleeding history (2 points), Reduced renal function (eGFR < 60 mL/min, 1 point) and Treatment with an antiplatelet (1 point)) [25] (Table S2). The bleeding risk of patients was categorised as either low (ORBIT score 0–2), medium (ORBIT score 3) or high (ORBIT score ≥ 4).

### 2.5. Statistical Analysis

Descriptive statistics were used to summarise patient demographic and clinical characteristics. Categorical variables were summarised using frequency counts and percentages, and means with standard deviations (SD) for continuous variables. The proportion of patients who persisted with OAC therapy and those who discontinued was calculated. Chi-square and *t*-tests were used to compare categorical and continuous variables, respectively. The difference in discontinuation rate between OACs was explored using a multivariable Cox proportional hazard. The variables included in the model were age, sex, rurality, index year, eGFR and the comorbidities listed in Table 1.

A binary logistic regression was used to identify factors associated with OAC discontinuation using a stepwise backward elimination at a threshold *p*-value of 0.1. Variables with a *p*-value < 0.1 were included in the final multivariable logistic regression model. Multicollinearity among variables was checked using a variance inflation factor (VIF). The absence of collinearity was declared if VIF was less than five. The CHA_2_DS_2_-VASc and ORBIT scores were not included in the model to minimise structural collinearity with individual components of the scores. Data were analysed using SAS version 9.4 (SAS Institute Inc., Cary, NC, USA), and statistical significance was considered at a two-sided *p*-value < 0.05 with a 95% confidence interval (CI).

## 3. Results

### 3.1. Patient Characteristics

In total, 16,075 patients with AF (47.3% females and a mean age of 74.6 (SD 10.2) years) who were newly prescribed an OAC were included in the study. Warfarin was prescribed to 27.9% (*n* = 4490), apixaban to 29.6% (*n* = 4761), dabigatran to 10.3% (*n* = 1651) and rivaroxaban to 32.2% (*n* = 5173) patients. Hypertension and arthritis were the most common comorbidities recorded, in 70.9% and 60.8% of patients, respectively. Most of the patients (91.2%) had a CHA_2_DS_2_-VASc score ≥ 2, with a mean value of 3.9 (SD 1.9), while the baseline bleeding risk was low in 66.3% of patients, with a mean ORBIT score of 1.9 (SD 1.4) (Table 1).

### 3.2. Discontinuation of OAC Therapy

**Figure 1:**
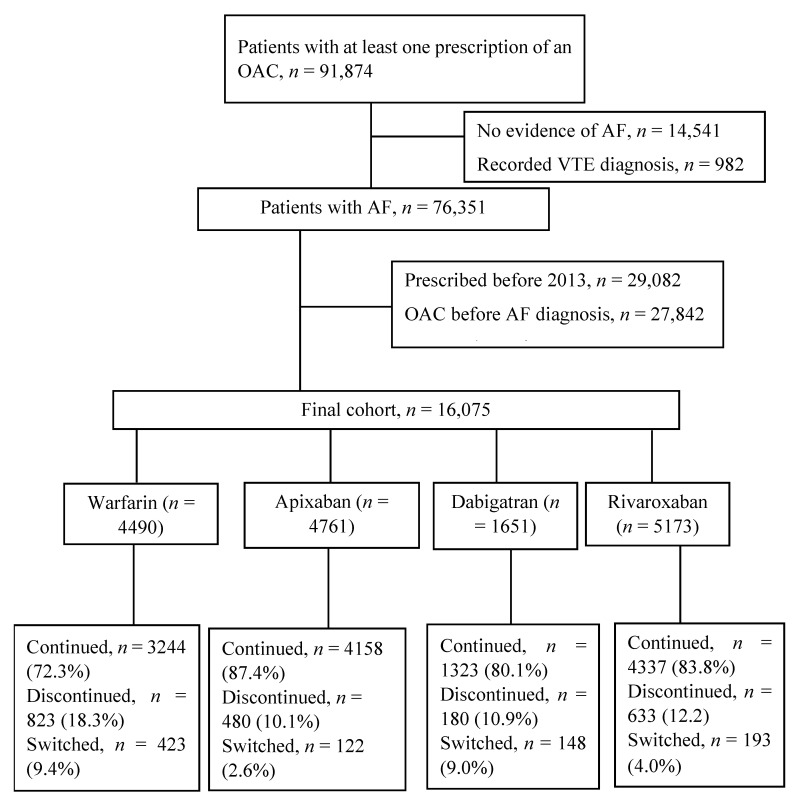
Flowchart of the cohort selection process (AF: atrial fibrillation, OAC: oral anticoagulant; VTE: venous thromboembolism).

Of the total patients included in the analysis, 2116 (13.2%; 95% confidence interval (CI) 12.6–13.7%) discontinued their therapy within a year of initiation. The rates of discontinuation were 18.3% (95% CI 17.2–19.5%) for warfarin users and 11.2% (95% CI 10.6–11.8%) for DOAC users (*p* < 0.001). Among patients who initiated apixaban, dabigatran and rivaroxaban, 10.1% (95% CI 9.2–11.0%), 10.9% (95% CI 9.4–12.5%) and 12.2% (95% CI 11.4–13.2%) discontinued their OAC by the end of one year, respectively (Figure 1).

The majority of OAC discontinuation (69.3%) occurred within six months post-initiation. At 6 and 12 months, persistence with an OAC averaged 90.9% (95% CI 90.4–91.3%) and 86.8% (95% CI 86.3–87.4%), respectively.

### 3.3. Predictors of OAC Discontinuation

On multivariable logistic regression, variables associated with better persistence with OAC were older age, and comorbidities such as hypertension, diabetes, and arthritis. Patients who started on warfarin were more likely to discontinue (AOR 1.70; 95% CI 1.39–2.08) compared to those patients who initiated dabigatran (Table 2).

We also performed a Cox proportional hazard to compare the risk of discontinuation between OACs by adjusting baseline differences. Accordingly, the discontinuation rate of warfarin was significantly higher than the three DOACs: apixaban (hazard ratio (HR) 1.78; 95% CI 1.54–2.06), dabigatran (HR 1.56; 95% CI 1.29–1.87) and rivaroxaban (HR 1.52; 95% CI 1.33–1.73). Rivaroxaban had also a slightly increased risk of discontinuation compared to apixaban (HR 1.17; 95% CI 1.03–1.34). However, the rate of dabigatran discontinuation did not show a statistically significant difference compared to apixaban (HR 1.14; 95% CI 0.94–1.39) or rivaroxaban (HR 0.98; 95% CI 0.81–1.17).

### 3.4. Sensitivity Analyses

We conducted two sensitivity analyses to examine the effect of changing the maximum allowable gap days from 60 to 30 days and 60 to 90 days to define discontinuation. As expected, there was an increase in the discontinuation rate using a gap of 30 days (15.1% vs. 13.2%), with decreased discontinuation using 90 days (11.4% vs. 13.2%) relative to 60 days. These relatively small changes in the rates of discontinuation did not significantly affect the comparative risk of discontinuation between OACs in the Cox proportional model (Table S3) and had minimal influence on the predictors of overall OAC discontinuation (Tables S4 and S5).

## 4. Discussion

In this retrospective cohort of 16,075 patients with AF who newly initiated either warfarin or a DOAC, more than one in eight patients discontinued their therapy by the end of 12 months post-initiation. Only a few studies from Australia, conducted using PBS data are available for comparison [13,14], reporting higher discontinuation rates of 30% and 62% at 12 months for DOACs and warfarin, respectively. It might be difficult to make a direct comparison as the latter studies were conducted in an earlier period following the market authorisation of DOACs in Australia, as well as using a different data source (prescription claims data). It is presumably reasonable that studies using prescription claims data sources would report a higher discontinuation rate given that the data source combines patients who failed to continue receiving prescriptions as well as those who did not continue to refill prescriptions from a pharmacy.

On other hand, there are some studies around the world conducted using prescription data from general practices. These include ones from UK, Germany, and France (1-year non-persistence rate: 34.1%; 42.6% and 57.5%, respectively) [26,27,28]. Several reasons could explain the higher non-persistence rate in those studies. Firstly, in the aforementioned studies, switching to another OAC was considered as discontinuation. Secondly, key differences in the characteristics of patients were also evident in our and the prior studies, which could influence therapy persistence. In comparison to the current study, a higher proportion of patients had a relatively low risk of stroke and high bleeding risk score in the previous studies. Both factors were implicated here in poor persistence. Moreover, most of the discontinuation in the previous studies was driven by vitamin K antagonists, while low proportions of patients initiated DOACs, which we and others have found are usually associated with better persistence [29]. Warfarin discontinuation was higher than for DOACs (i.e., 18.3% vs. 11.2%). The difference remained significant after adjusting for confounders and at a discontinuation gap of 30 and 90 days. A study conducted using a prescription claims dataset also found higher discontinuation in warfarin users (62% vs. 30% of DOACs) [14]. Several previous studies also reported a higher rate of warfarin discontinuation compared to each DOAC [30]. This could be related to either incident bleeding events or anticipated higher risk of bleeding with warfarin or the risk of frailty in the elderly [31].

Another key finding was the timing of the majority discontinuation; more than two-thirds occurred during the first six months of treatment, particularly for DOACs. This signifies a need for more regular follow-up during the immediate post-initiation period [32]. In the warfarin treatment group, a significant proportion of discontinuation came after six months, perhaps associated with patients starting to give up therapy due to the inconveniences associated with the drug. Similar findings were reported in Germany, where patients who were prescribed a vitamin K antagonist showed better persistence in the early months after initiation, followed by a subsequent rise in non-persistence beyond three months post-initiation [27].

Among DOACs, the rate of discontinuation was comparable. Rivaroxaban had a slightly higher hazard of discontinuation (HR 1.17; 95% CI 1.03–1.34) than apixaban, in line with a study from the United States (HR 1.12; 95% CI 1.09–1.15) [33] and the UK (OR 1.18; 95% CI 1.08–1.30) [34]. It would be assumed that once-daily dosing of rivaroxaban could positively contribute to improved persistence than twice-daily apixaban [35]. However, there are complex factors such as patient characteristics, bleeding risks and relative effectiveness [18,36], beyond dosing frequency, which would influence the probability of discontinuing an OAC. Nevertheless, the difference in the discontinuation rate of dabigatran was not statistically significant compared to apixaban and rivaroxaban. In several studies that used prescription data [26,27,28,34], dabigatran was associated with higher discontinuation compared to other DOACs. However, those studies usually considered switching, which is more prevalent among dabigatran users [37,38,39], as discontinuation.

Our findings are in line with previous studies showing factors that increase the risk of stroke were associated with better OAC persistence [27,34,40,41,42]. Older patients (>65 years), and those diagnosed with hypertension and diabetes at baseline had lower odds of discontinuation. A positive correlation between age and medication-taking behaviour in general and persistence, in particular, is widely cited [18,34,40]. Patients with comorbidities and the highest risk of stroke could be more aware of the dangers associated with discontinuation, besides having more close monitoring and frequent follow-up that would help to improve persistence. In addition, in low-risk patients, the clinicians might decide to discontinue the OAC as the risks associated with therapy could outweigh the clinical benefits [5]. Patients with arthritis had better persistence. This may be partly because they might visit their doctor more frequently to manage arthritic pain, creating an opportunity for clinicians to closely monitor OAC therapy and improve persistence.

The study has some limitations to be considered when interpreting the findings. First, the definition of warfarin exposure is an approximation by assuming a defined daily dose. This is because the dataset lacks INR records that could be helpful to measure warfarin exposure. This may produce a bias, and it is difficult to be certain of the comparison with DOACs. Second, our study used prescribing data, which could not show whether the patient obtained the medication and took it as prescribed. Third, there is a possibility that patients may change their practice site and visit a general practice not registered in MedicineInsight, which may lead to the misclassification of patients as discontinued. To minimise this, we included patients who were regular attendees to a practice site and checked the presence of a visit to a single practice site after the patient was classified as discontinued. Fourth, the dataset captured prescriptions originating from general practices, while prescriptions from hospitals and specialist clinics were not included, and no linkage was available. Finally, the dataset did not provide data on the reasons for discontinuation and clinical outcomes following the discontinuation of OAC.

## 5. Conclusions

The study demonstrated two key findings: a relatively low proportion of patients discontinued their OAC within 12 months post-initiation, and patients at the highest risk of stroke and lowest risk of bleeds seemed to have the highest persistence. Although there was a high persistence rate with OAC therapy, patient education would be helpful to further enhance the long-term continuity of therapy, especially in younger patients and those taking warfarin. Further study is needed to understand the reasons for discontinuation. Additionally, using linked data with prescription claims databases and hospital records could provide complete data to investigate medication possession and the clinical outcomes of discontinuation.

## Figures and Tables

**Table 1:** Baseline characteristics of study participants.

Variables	All Patients (%) (*n* = 16,075)	Persisted (%) (*n* = 13,959)	Discontinued (%) (*n* = 2116)	*p*-Value
Follow up in days, Mean (SD)	329.7 (79.1)	348.9 (64.3)	202.9 (42.0)	<0.001
Sex, female	7602 (47.3)	6642 (47.6)	960 (45.4)	0.057
Age, mean (SD), years	74.6 (10.2)	74.8 (9.8)	73.4 (12.4)	<0.001
Age group				<0.001
<65 years	2439 (15.2)	1981 (14.2)	458 (21.6)	
65–74 years	5002 (31.1)	4421 (31.7)	581 (27.5)	
≥75 years	8634 (53.7)	7557 (54.1)	1077 (50.9)	
Index year				<0.001
2013	2629 (16.4)	2175 (15.6)	454 (21.5)	
2014	2986 (18.6)	2590 (18.6)	396 (18.7)	
2015	3159 (19.6)	2759 (19.8)	400 (18.9)	
2016	3431 (21.3)	3027 (21.7)	404 (19.1)	
2017	3870 (24.1)	3408 (24.4)	462 (21.8)	
ATSI	*n* = 13,542	*n* = 11,828	*n* = 1714	0.72
Yes	177 (1.3)	153 (1.3)	24 (1.4)	
No	13,365 (98.7)	11,675 (98.7)	1690 (98.6)	
Rurality	*n* = 15,994	*n* = 13,897	*n* = 2097	<0.001
Major cities	9452 (59.1)	8225 (59.2)	1227 (58.5)	
Inner regional	4863 (30.4)	4270 (30.7)	593 (28.3)	
Outer regional	1444 (9.0)	1204 (8.7)	240 (11.4)	
Remote/very remote	235 (1.5)	198 (1.4)	37 (1.8)	
SEIFA quintiles	*n* = 15,975	*n* = 13,750	*n* = 2225	0.18
1	2626 (16.4)	2239 (16.3)	387 (17.4)	
2	3418 (21.4)	2956 (21.5)	462 (20.8)	
3	4029 (25.2)	3505 (25.5)	524 (23.6)	
4	2724 (17.0)	2338 (17.0)	386 (17.3)	
5	3178 (19.9)	2712 (19.5)	466 (20.9)	
Comorbidities				
Hypertension	11,397 (70.9)	10,045 (72.0)	1352 (63.9)	<0.001
Heart failure	4206 (26.2)	3694 (26.5)	512 (24.2)	0.027
Diabetes	4080 (25.4)	3609 (25.8)	471 (22.3)	<0.001
Stroke	3135 (19.5)	2760 (19.8)	375 (17.7)	0.027
Vascular disease	6187 (38.5)	5391(38.6)	796 (37.6)	0.38
Anxiety	2802 (17.4)	2426 (17.4)	376 (17.8)	0.66
Arthritis	9768 (60.8)	8628 (61.8)	1140 (53.9)	<0.001
Asthma	2959 (18.4)	2594 (18.6)	365 (17.2)	0.14
COPD	2721 (16.9)	2365 (16.9)	356 (16.8)	0.89
Depression	4015 (25.0)	3510 (25.2)	505 (23.9)	0.20
Dementia	724 (4.5)	621 (4.4)	103 (4.9)	0.39
eGFR in mL/min	*n* = 12,736	*n* = 11,135	*n* = 1601	0.16
≥60	6752 (53.0)	5886 (52.9)	866 (54.1)	
45–59	2992 (23.5)	2635 (23.7)	357 (22.3)	
30–44	2027 (15.9)	1787 (16.0)	240 (15.0)	
<30	965 (7.6)	827 (7.4)	138 (8.6)	
CHA_2_DS_2_-VASc risk score				
Mean (SD)	3.9 (1.8)	3.9 (1.7)	3.6 (2.0)	<0.001
0	558 (3.5)	364 (2.6)	194 (9.2)	<0.001
1	853 (5.3)	695 (5.0)	158 (7.5)	
≥2	14,664 (91.2)	12,900 (92.4)	1764 (83.4)	
ORBIT risk				
Mean (SD)	1.9 (1.4)	1.9 (1.4)	2.1 (1.5)	<0.001
Low	10,661 (66.3)	9351 (67.0)	1310 (61.9)	<0.001
Medium	2991 (18.6)	2568 (18.4)	423 (20.0)	
High	2423 (15.1)	2040 (14.6)	383 (18.1)	

ATSI: Aboriginal and Torres Strait Islander; COPD: chronic obstructive pulmonary disease; eGFR: estimated glomerular filtration rate; SD: standard deviation; SEIFA: socioeconomic index for area.

**Table 2:** Factors associated with discontinuation of OACs.

	AOR (95% CI)	*p*-Value
Age category		
<65 years	Ref	
65–74 years	0.60 (0.52–0.71)	<0.001
≥75 years	0.68 (0.56–0.76)	<0.001
Rurality		
Major cities	0.94 (0.60–1.52)	0.78
Inner regional	0.85 (0.54–1.38)	0.49
Outer regional	1.26 (0.80–2.04)	0.33
Remote/very remote	Ref	
Comorbidities		
Hypertension	0.73 (0.65–0.82)	<0.001
Diabetes	0.88 (0.77–0.99)	0.043
Stroke	0.94 (0.88–1.01)	0.081
Arthritis	0.81 (0.72–0.90)	0.0001
Anxiety	1.13 (0.98–1.29)	0.086
OAC		
Dabigatran	Ref	
Warfarin	1.70 (1.39–2.08)	<0.001
Rivaroxaban	1.06 (0.87–1.30)	0.54
Apixaban	0.90 (0.73–1.10)	0.30

## Data Availability

Data sharing is restricted by the owner of the dataset; thus, data other than those included in the manuscript will not publicly available. Access to the dataset should be requested directly from the data owner through datagovernance@nps.org.au.

## Supplementary Materials

The following supporting information can be downloaded at: www.mdpi.com/article/10.3390/jcm11206022/s1, Table S1: Coded and non-coded terms used to identify medical conditions from the MedicineInsight dataset [23], Table S2: Definition and scoring of stroke and bleeding risks [24,25], Table S3: Cox proportional hazard of discontinuation at different days gap, Table S4: Predictors of OAC discontinuation at 30 days of discontinuation gap, Table S5: Predictors of OAC discontinuation at 90 days of discontinuation gap.

## References

[1] Pistoia F., Sacco S., Tiseo C., Degan D., Ornello R., Carolei A. (2016). The Epidemiology of Atrial Fibrillation and Stroke. Cardiol. Clin..

[2] Wodchis W.P., Bhatia R.S., Leblanc K., Meshkat N., Morra D. (2012). A Review of the Cost of Atrial Fibrillation. Value Health.

[3] Roth G.A., Mensah G.A., Johnson C.O., Addolorato G., Ammirati E., Baddour L.M., Barengo N.C., Beaton A.Z., Benjamin E.J., Benziger C.P. (2020). Global Burden of Cardiovascular Diseases and Risk Factors, 1990–2019: Update From the GBD 2019 Study. J. Am. Coll. Cardiol..

[4] (2019). Global Burden of Disease Study 2019 (GBD 2019).

[5] Kirchhof P., Benussi S., Kotecha D., Ahlsson A., Atar D., Casadei B., Castella M., Diener H.-C., Heidbuchel H., Hendriks J. (2016). 2016 ESC Guidelines for the management of atrial fibrillation developed in collaboration with EACTS. Eur. Heart J..

[6] Hart R.G., Pearce L., Aguilar M.I. (2007). Meta-analysis: Antithrombotic Therapy to Prevent Stroke in Patients Who Have Nonvalvular Atrial Fibrillation. Ann. Intern. Med..

[7] Ruff C.T., Giugliano R.P., Braunwald E., Hoffman E.B., Deenadayalu N., Ezekowitz M.D., Camm A.J., Weitz J.I., Lewis B.S., Parkhomenko A. (2014). Comparison of the efficacy and safety of new oral anticoagulants with warfarin in patients with atrial fibrillation: A meta-analysis of randomised trials. Lancet.

[8] Miller C.S., Grandi S.M., Shimony A., Filion K.B., Eisenberg M.J. (2012). Meta-Analysis of Efficacy and Safety of New Oral Anticoagulants (Dabigatran, Rivaroxaban, Apixaban) Versus Warfarin in Patients With Atrial Fibrillation. Am. J. Cardiol..

[9] Loffredo L., Perri L., Violi F. (2015). Impact of new oral anticoagulants on gastrointestinal bleeding in atrial fibrillation: A meta-analysis of interventional trials. Dig. Liver Dis..

[10] Bezabhe W.M., Bereznicki L.R., Radford J., Wimmer B.C., Curtain C., Salahudeen M.S., Peterson G.M. (2021). Ten-Year Trends in the Use of Oral Anticoagulants in Australian General Practice Patients With Atrial Fibrillation. Front. Pharmacol..

[11] Bezabhe W.M., Bereznicki L.R., Radford J., Wimmer B.C., Curtain C., Salahudeen M.S., Peterson G.M. (2020). Factors influencing oral anticoagulant use in patients newly diagnosed with atrial fibrillation. Eur. J. Clin. Investig..

[12] Komen J.J., Heerdink E.R., Klungel O.H., Mantel-Teeuwisse A.K., Forslund T., Wettermark B., Hjemdahl P. (2021). Long-term persistence and adherence with non-vitamin K oral anticoagulants in patients with atrial fibrillation and their associations with stroke risk. Eur. Hearth J. Cardiovasc. Pharmacother..

[13] Simons L.A., Ortiz M., Freedman B., Waterhouse B.J., Colquhoun D. (2017). Medium- to long-term persistence with non-vitamin-K oral anticoagulants in patients with atrial fibrillation: Australian experience. Curr. Med. Res. Opin..

[14] Simons L.A., Ortiz M., Freedman B., Waterhouse B.J., Colquhoun D., Thomas G. (2016). Improved persistence with non-vitamin-K oral anticoagulants compared with warfarin in patients with atrial fibrillation: Recent Australian experience. Curr. Med. Res. Opin..

[15] (2021). MedicineInsight Data Book Version 4.

[16] Busingye D., Gianacas C., Pollack A., Chidwick K., Merrifield A., Norman S., Mullin B., Hayhurst R., Blogg S., Havard A. (2019). Data Resource Profile: MedicineInsight, an Australian national primary health care database. Int. J. Epidemiology.

[17] Youens D., Moorin R., Harrison A., Varhol R., Robinson S., Brooks C., Boyd J. (2020). Using general practice clinical information system data for research: The case in Australia. Int. J. Popul. Data Sci..

[18] Hohnloser S.H., Basic E., Nabauer M. (2019). Changes in Oral Anticoagulation Therapy over One Year in 51,000 Atrial Fibrillation Patients at Risk for Stroke: A Practice-Derived Study. Thromb. Haemost..

[19] The Royal Australian College of General Practitioners (2020). Standards for General Practices.

[20] Maura G., Billionnet C., Alla F., Gagne J.J., Pariente A. (2017). Comparison of Treatment Persistence with Dabigatran or Rivaroxaban versus Vitamin K Antagonist Oral Anticoagulants in Atrial Fibrillation Patients: A Competing Risk Analysis in the French National Health Care Databases. Pharmacother. J. Hum. Pharmacol. Drug Ther..

[21] Australian Bureau of Statistics (2019). Australian Statistical Geography Standard.

[22] Australian Bureau of Statistics (2016). Census of Population and Housing: Socio-Economic Indexes for Areas (SEIFA).

[23] Bezabhe W.M., Bereznicki L.R., Radford J., Wimmer B.C., Salahudeen M.S., Garrahy E., Bindoff I., Peterson G.M. (2022). Oral Anticoagulant Treatment and the Risk of Dementia in Patients With Atrial Fibrillation: A Population-Based Cohort Study. J. Am. Hearth Assoc..

[24] Lip G.Y., Nieuwlaat R., Pisters R., Lane D.A., Crijns H.J. (2010). Refining clinical risk stratification for predicting stroke and thromboembolism in atrial fibrillation using a novel risk factor-based approach: The euro heart survey on atrial fibrillation. Chest.

[25] O’Brien E.C., Simon D.N., Thomas L.E., Hylek E.M., Gersh B.J., Ansell J.E., Kowey P.R., Mahaffey K.W., Chang P., Fonarow G.C. (2015). The ORBIT bleeding score: A simple bedside score to assess bleeding risk in atrial fibrillation. Eur. Hearth J..

[26] Banerjee A., Benedetto V., Gichuru P., Burnell J., Antoniou S., Schilling R.J., Strain W.D., Ryan R., Watkins C., Marshall T. (2020). Adherence and persistence to direct oral anticoagulants in atrial fibrillation: A population-based study. Heart.

[27] Collings S.-L., Lefèvre C., Johnson M.E., Evans D., Hack G., Stynes G., Maguire A. (2017). Oral anticoagulant persistence in patients with non-valvular atrial fibrillation: A cohort study using primary care data in Germany. PLoS ONE.

[28] Collings S.-L., Vannier-Moreau V., Johnson M.E., Stynes G., Lefèvre C., Maguire A., Asmar J., Bizouard G., Duhot D., Mouquet F. (2018). Initiation and continuation of oral anticoagulant prescriptions for stroke prevention in non-valvular atrial fibrillation: A cohort study in primary care in France. Arch. Cardiovasc. Dis..

[29] Ozaki A.F., Choi A.S., Le Q.T., Ko D.T., Han J.K., Park S.S., Jackevicius C.A. (2020). Real-World Adherence and Persistence to Direct Oral Anticoagulants in Patients with Atrial Fibrillation: A Systematic Review and Meta-Analysis. Circ. Cardiovasc. Qual Outcomes.

[30] Jackson L.R., Kim S., Blanco R., Thomas L., Ansell J., Fonarow G.C., Gersh B.J., Go A.S., Kowey P.R., Mahaffey K.W. (2020). Discontinuation rates of warfarin versus direct acting oral anticoagulants in US clinical practice: Results from Outcomes Registry for Better Informed Treatment of Atrial Fibrillation II (ORBIT-AF II). Am. Heart J..

[31] Buck J., Fromings Hill J., Martin A., Springate C., Ghosh B., Ashton R., Lee G., Orlowski A. (2021). Reasons for discontinuing oral anticoagulation therapy for atrial fibrillation: A systematic review. Age Ageing.

[32] Steffel J., Collins R., Antz M., Cornu P., Desteghe L., Haeusler K.G., Oldgren J., Reinecke H., Roldan-Schilling V., Rowell N. (2021). 2021 European Heart Rhythm Association Practical Guide on the Use of Non-Vitamin K Antagonist Oral Anticoagulants in Patients with Atrial Fibrillation. Europace.

[33] Baker C.L., Dhamane A.D., Mardekian J., Dina O., Russ C., Rosenblatt L., Lingohr-Smith M., Menges B., Lin J., Nadkarni A. (2019). Comparison of Drug Switching and Discontinuation Rates in Patients with Nonvalvular Atrial Fibrillation Treated with Direct Oral Anticoagulants in the United States. Adv. Ther..

[34] Ruigómez A., Vora P., Balabanova Y., Brobert G., Roberts L., Fatoba S., Fernandez O., Rodríguez L.A.G. (2019). Discontinuation of non-Vitamin K antagonist oral anticoagulants in patients with non-valvular atrial fibrillation: A population-based cohort study using primary care data from The Health Improvement Network in the UK. BMJ Open.

[35] Ageno W., Beyer-Westendorf J., Rubboli A. (2017). Once- versus twice-daily direct oral anticoagulants in non-valvular atrial fibrillation. Expert Opin. Pharmacother..

[36] Fralick M., Colacci M., Schneeweiss S., Huybrechts K.F., Lin K.J., Gagne J.J. (2020). Effectiveness and Safety of Apixaban Compared With Rivaroxaban for Patients With Atrial Fibrillation in Routine Practice: A Cohort Study. Ann. Intern. Med..

[37] Kefale A.T., Peterson G.M., Bezabhe W.M., Bereznicki L.R. (2022). Switching of oral anticoagulants in patients with non-valvular atrial fibrillation: A narrative review. Br. J. Clin. Pharmacol..

[38] Romoli M., Marchetti G., Bernardini F., Urbinati S. (2021). Switching between direct oral anticoagulants: A systematic review and me-ta-analysis. J. Thromb. Thrombolysis.

[39] Kefale A.T., Peterson G.M., Bezabhe W.M., Bereznicki L.R. (2022). Switching of oral anticoagulants in atrial fibrillation: A cohort study using Aus-tralian general practice data. Expert Rev. Clin. Pharmacol..

[40] Lowres N., Giskes K., Hespe C., Freedman B. (2019). Reducing Stroke Risk in Atrial Fibrillation: Adherence to Guidelines Has Improved, but Patient Persistence with Anticoagulant Therapy Remains Suboptimal. Korean Circ. J..

[41] Hellfritzsch M., Husted S.E., Grove E.L., Rasmussen L., Poulsen M.H., Johnsen S.P., Hallas J., Pottegård A. (2017). Treatment Changes among Users of Non-Vitamin K Antagonist Oral Anticoagulants in Atrial Fibrillation. Basic Clin. Pharmacol. Toxicol..

[42] Dhamane A.D., Hernandez I., Di Fusco M., Gutierrez C., Ferri M., Russ C., Tsai W.-L., Emir B., Yuce H., Keshishian A. (2022). Non-persistence to Oral Anticoagulation Treatment in Patients with Non-valvular Atrial Fibrillation in the USA. Am. J. Cardiovasc. Drugs.

