# Supplementary material for: Oral Anticoagulant Discontinuation and Its Predictors in Patients with Atrial Fibrillation
Source: J Clin Med. 2022 Oct 12;11(20):6022. doi: 10.3390/jcm11206022 (PMC9605276; doi:10.3390/jcm11206022)
Supplement: Supplementary file 1 [file jcm-11-06022-s001.zip › jcm-1948158-supplementary.pdf]

Supplementary material

Table S1: Coded and non-coded terms used to identify medical conditions from the MedicineInsight dataset [23]

Comorbid condition	Terms used to identify from the dataset
Atrial fibrillation	AF; AF (Atrial Fibrillation); Arrhythmia, Atrial Fibrillation; Atrial Fibrillation; Atrial Fibrillation - Isolated Episode; Atrial Fibrillation - Paroxysmal; Atrial Fibrillation, Non-Valvular; Atrial Fibrillation, Valvular; Fibrillation – Atrial; Fibrillation Atrium - Paroxysmal; Fibrillation, Atrial; Non-Valvular Atrial Fibrillation; Paroxysmal Atrial Fibrillation; Rapid AF; Rapid Atrial Fibrillation; Valvular Atrial Fibrillation
Anxiety	Adjustment Disorder with Anxiety; Adjustment Disorder with Mixed Anxiety and Depressed Mood; Anxiety; Anxiety-Generalised; Anxiety – PTSD; Anxiety – Social; Anxiety Disorder; Anxiety Disorder, Substance Induced; Anxiety Neurosis; Anxiety Phobia; Anxiety With Panic Attacks; Anxiety/Depression; Depression/Anxiety; Depressive Anxiety Disorder; GAD; GAD (Generalised Anxiety Disorder); Generalised Anxiety Disorder; Generalised Anxiety Disorder (GAD); Mixed Anxiety Depression; Mixed Anxiety/Depressive Disorder; Mixed Depression Anxiety; Nervous Anxiety; Neurotic Anxiety; Phobic Anxiety Disorder; Social Anxiety Disorder; Social Phobia; Substance Induced Anxiety Disorder
Arthritis	Ac Joint Arthritis; Acromioclavicular Joint Arthritis; Aneurysm-Osteoarthritis Syndrome; Ankle Osteoarthritis; Ankylosing Spondylitis; Arthritis; Arthritis – Gouty; Arthritis – Juvenile Rheumatoid; Arthritis Lisfranc; Arthritis Lupus; Arthritis Osteo; Arthritis Psoriatic; Arthritis Rheumatoid; Arthritis – Septic; Arthritis – Seronegative; Arthritis – Viral; Arthritis Of Spine; Arthritis Of The Acromioclavicular Joint; Arthritis, Inflammatory; Arthritis, Juvenile Rheumatoid; Arthritis, Psoriatic; Arthritis, Rheumatoid; Arthritis, Septic; Arthritis, Seronegative; Arthritis, Viral; Caplan Syndrome; Cervical - Osteo Arthritis; Cervical Spine Osteoarthritis; Elbow Osteoarthritis; Facet Joint Arthritis; Generalised Osteoarthritis; Giant Cell Reticulohistiocytosis; Gout; Gouty Arthritis; Hallux Rigidus; Hip Osteoarthritis; Hip Osteoarthrosis; Hyperuricaemia; Hyperuricemia; Inflammatory Polyarthritis; Joint Infection; Jra; Jra (Juvenile Rheumatoid Arthritis); Juvenile Idiopathic Arthritis; Juvenile Rheumatoid Arthritis; Knee Osteoarthritis; Knee Osteoarthrosis; Lipoid Dermatoarthritis; Lipoid Rheumatism; Lisfranc Arthritis; Loeys-Dietz Syndrome Type 3; Lumbar Osteo Arthritis; Lumbar Spine Osteoarthritis; Lupus Arthritis; Lyme Arthritis; Midfoot Osteoarthritis; Monoarthritis; Multicentric Reticulohistiocytosis; Oa; Oa (Osteoarthritis); Oligoarthritis, Inflammatory; Osteoarthritis; Osteoarthritis – Ankle; Osteoarthritis – Elbow; Osteoarthritis – Fingers; Osteoarthritis Glenohumeral Joint; Osteoarthritis – Hands; Osteoarthritis – Hip; Osteoarthritis – Knee; Osteoarthritis – Neck; Osteoarthritis – Shoulder;

	Osteoarthritis – Spine; Osteoarthritis of 1st Carpometacarpal Joint; Osteoarthritis of 1st CarpoMetacarpal Joint; Osteoarthritis of 1st Metatarsophalangeal Joint; Osteoarthritis of Ankle; Osteoarthritis of Cervical Spine; Osteoarthritis of Elbow; Osteoarthritis of Fingers; Osteoarthritis of Foot; Osteoarthritis Of Hand; Osteoarthritis of Hip; Osteoarthritis of Knee; Osteoarthritis of Lumbar Spine; Osteoarthritis of Neck; Osteoarthritis of Sacroiliac Joints; Osteoarthritis of Shoulder; Osteoarthritis of The Patellofemoral Joint; Osteoarthritis of Thoracic Spine; Osteoarthritis of Tmj; Osteoarthritis of Wrist; Osteoarthritis Generalised; Osteoarthrosis; Patellofemoral Osteoarthritis; Podagra; Polyarthritis; Polyarthritis, Inflammatory; Psoriatic Arthritis; Psoriatic Arthropathy; RA; RA (Rheumatoid Arthritis); Reactive Arthritis; Reiter's Disease; Reiter's Syndrome; Rheumatoid Arthriti; Rheumatoid Arthritis Juvenile; Rheumatoid Arthritis – Pneumoconiosis; Rheumatoid Arthritis, Juvenile; Sacroiliac Joint Arthritis; Septic Arthritis; Seronegative Arthritis; Seronegative Rheumatoid Arthritis; Shoulder Osteoarthritis; Spondyloarthritis; Spondylosis; Stills Disease; Thoracic - Osteo Arthritis; Urate Crystal Deposition; Venereal Arthritis; Viral Arthritis - Waelsch's Syndrome; Wear And Tear Arthritis; Wrist Osteoarthritis
Asthma	Acute Severe Asthma; Allergic Asthma; Allergy Induced Asthma; Aspirin Sensitive Asthma; Asthma; Asthma - Allergy Induced; Asthma - Chronic Persistent; Asthma - Exercise Induced; Asthma - Frequent Episodic; Asthma Infective Exacerbation; Asthma - Infrequent Episodic; Asthma - Precipitated By Bacterial Infection; Asthma - Precipitated By Viral Infection; Asthma Action Plan; Asthma Action Plan Performed; Asthma Action Plan Printed; Asthma Care Plan; Asthma Care Plan Review; Asthma Cycle Of Care; Asthma Exacerbation; Asthma Review; Asthma, Allergic; Asthma, Allergy Induced; Asthma, Childhood; Asthma, Exercise Induced; Asthma, Frequent Episodic; Asthma, Infective Exacerbation; Asthma, Infrequent Episodic; Asthma, Occupational; Asthma, Thunderstorm; Bronchial Asthma; Care Plan, Asthma; Check Up, Asthma; Exercise Induced Asthma; Exertional Asthma; Frequent Episodic Asthma; Infective Exacerbation Of Asthma; Infrequent Episodic Asthma; Occupational Asthma; Review – Asthma; Samter's Triad; Status Asthmaticus; Thunderstorm Asthma; Wheezy Bronchitis
Congestive heart failure	Acute Cardiac Failure; Acute Heart Failure; Biventricular Heart Failure; Cardiac Failure; Cardiac Failure, Acute; CCF; Chronic Heart Failure; Congestive Cardiac Failure; Congestive Heart Failure; Cor Pulmonale; Diastolic Cardiac Dysfunction; Diastolic Heart Failure; Heart Failure; Heart Failure – Acute; Heart Failure – Biventricular; Heart Failure - Chronic ; Heart Failure - High Output; Heart Failure – Left; Heart Failure - Mid Range Ejection Fraction; Heart Failure - Preserved Ejection Fraction; Heart Failure - Reduced Ejection Fraction; Heart Failure – Right; Heart Failure, Acute; Heart Failure, High Output; Heart Failure, Left; HFMREF; HFPEF; HFREF; High Output Cardiac Failure; High Output Heart Failure;

	Hypertensive Heart Failure; Left Heart Failure; Left Ventricular Failure; LHF; LHF (Left Heart Failure); LVF; LVF (Left Ventricular Failure); Pulmonary Oedema; RHF; RHF (Right Heart Failure); Right Heart Failure; Right Ventricular Failure; RVF; RVF (Right Ventricular Failure); Systolic Cardiac Dysfunction; Systolic Heart Failure; Ventricular Diastolic Dysfunction
COPD	Acute Exacerbation of COPD; Bronchitis – Chronic; Bronchitis, Chronic; CAL (Chronic Airways Limitation); Chronic Airways Limitation; Chronic Bronchitis; Chronic Bronchitis - Infective Exacerbation; Chronic Bronchitis, Infective Exacerbation; Chronic Obstructive Airways Disease; Chronic Obstructive Pulmonary Disease; COAD; COAD - Infective Exacerbation; COAD (Chronic Obstructive Airways Disease); COAD, Infective Exacerbation; COPD; COPD -Infective Exacerbation; COPD (Chronic Obstructive Pulmonary Disease); COPD, Infective Exacerbation; Emphysema; Emphysema - Infective Exacerbation; Infective Exacerbation of Chronic Bronchitis; Infective Exacerbation of COAD; Infective Exacerbation of COPD
Dementia	Alzheimer's Disease; Binswanger Disease; Binswanger's Encephalopathy; Dementia; Dementia – Frontotemporal; Dementia - Lewy-Body; Dementia - Multi Infarct; Dementia – Pick; Dementia – Vascular; Dementia Related Psychosis; Dementia With Lewy Bodies; Dementia, Early Onset; Dementia, Frontotemporal; Dementia, Multi Infarct; Dementia, Pick's; Dementia, Semantic; Dementia, Substance Induced; Dementia, Vascular; Early Onset Dementia; Frontotemporal Dementia; Korsakoff's Dementia; Korsakoff's Psychosis; Korsakov's Psychosis; Lewy Body Dementia; Major Neurocognitive Disorder Due To Alzheimer's Disease; Multi Infarct Dementia; Neurocognitive Disorder, Major, Due To Alzheimer's Disease; Parkinson's Disease - Lewy Body Dementia; Pick's Disease; Psychosis - Korsakoff's; Psychosis, Dementia Related; Semantic Dementia; Senile Dementia With Psychosis; Subcortical Arteriosclerotic Encephalopathy; Subcortical Dementia; Substance Induced Dementia; Vascular Dementia; Young Onset Dementia
Depression	Adjustment Disorder (Chronic) With Depressed And Anxious Mood; Adjustment Disorder (Chronic) With Depressed Mood; Adjustment Disorder With Depressed And Anxious Mood; Adjustment Disorder With Mixed Anxiety And Depressed Mood; Anxiety/Depression; Chronic Adjustment Disorder With Depressed And Anxious Mood; Chronic Adjustment Disorder With Depressed Mood; Depression; Depression – Endogenous; Depression – Minor; Depression - Post Natal; Depression – Reactive; Depression – Recurrent; Depression – Subsyndromal; Depression With Melancholic Features; Depression, Endogenous; Depression, Melancholic; Depression, Non Melancholic; Depression, Organic; Depression, Postnatal; Depression, Psychotic; Depression, Reactive; Depression/Anxiety; Depressive Anxiety Disorder; Depressive Episode, Major; Endogenous Depression; Insomnia - Depression-Related; Involutional Melancholia; Major Depression; Major

	Depressive Episode; Melancholia; Melancholia – Involutional; Melancholic Depression; Mixed Anxiety Depression; Mixed Anxiety/Depressive Disorder; Mixed Depression Anxiety; Neurotic Depression; Non Melancholic Depression; Organic Depression; Post Natal Depression; Postnatal Depression; Psychotic Depression; Reactive Depression
Diabetes mellitus	Diabetes Mellitus – IDDM; Diabetes Mellitus - Type I; Diabetes Mellitus, IDDM; Diabetes Mellitus, Type 1; IDDM; IDDM (Insulin Dependent Diabetes Mellitus); Insulin Dependent Diabetes Mellitus; Juvenile Onset Diabetes; Juvenile Onset Diabetes Mellitus; Diabetes Mellitus – NIDDM; Diabetes Mellitus - Type II; Diabetes Mellitus, NIDDM; Diabetes Mellitus, Type 2; Diabetes Type II Requiring Insulin; NIDDM; NIDDM - Requiring Insulin; NIDDM (Non-Insulin Dependent Diabetes Mellitus); Non-Insulin Dependent Diabetes Mellitus; T2DM; Type 2 Diabetes Mellitus
Hypertension	Hypertension – Controlled; Essential Hypertension; HBP; High Blood Pressure; HT (Hypertension); Hypertension; Primary Hypertension; Hypertension – Malignant; Malignant Hypertension; Severe Refractory Hypertension; Hypertension – Pregnancy; PIH; Pregnancy Induced Hypertension; Hypertension – Renovascular; Renal Hypertension; Renovascular Hypertension; Hypertension Isolated Systolic; Blood Pressure Labile; BP Labile; BP Unstable; Hypertension – Labile; Hypertension – Unstable; Labile Blood Pressure; Labile BP; Labile Hypertension; Hypertension - Life Style Management; Antihypertensive Agent Prescription; Blood Pressure Review; Hypertension Review; Review – BP; Hypertension, Essential; Hypertension, Malignant; Hypertension In Pregnancy; Hypertension, Renovascular; Hypertension, Isolated Systolic; Isolated Systolic Hypertension; Diastolic Hypertension; Hypertension, Diastolic
Stroke	Cerebral Haemorrhage; Cerebral Infarction; Cerebrovascular Accident; CVA; CVA (Cerebrovascular Accident); Haemorrhage – Intracerebral; Haemorrhage, Intracerebral; Haemorrhagic CVA; Haemorrhagic Stroke; Intracerebral Bleed; Intracerebral Haemorrhage; Intracranial Haemorrhage; Ischaemic Stroke; Lacunar Infarct; Lacunar Stroke; Migrainous Stroke; Migranous Stroke; Stroke; Stroke – Haemorrhagic; Stroke – Ischaemic; Stroke – Lacunar; Stroke – Migranous; Stroke – Thrombotic; Stroke, Haemorrhagic; Stroke, Ischaemic; Stroke, Lacunar; Stroke, Migrainous; Stroke, Thrombotic; Thrombotic – Stroke; Thrombotic Stroke; Visual Cortex Stroke
Vascular disease	Arteriosclerosis Obliterans; Arteritis - Diabetes Mellitus; Buerger's Disease; Diabetes With Vascular Changes; Diabetic Endarteritis; Diabetic Peripheral Vascular Disease; Diabetic Vascular Disease – Peripheral; Obliterative Vascular Disease; Occlusive Vascular Disease; Occlusive Vascular Disease (Buerger's Disease); Peripheral Arterial Disease; Peripheral Arterial Occlusive Disease (Buerger's Disease); Peripheral Vascular Disease; Peripheral Vascular Disease, Diabetic; PVD; Thrombangiitis Obliterans; Thromboangiitis Obliterans

Table S2: Definition and scoring of stroke and bleeding risks [24, 25].

**Tool components**	**Definition**	**Score**
1. **CHA_2DS_2-VASc**		
Congestive heart failure	Recent signs, symptoms, or admission for decompensated heart failure; this includes both reduced and preserved ejection fraction heart failure, or moderately to severely reduced systolic left ventricular function, whether or not there is a history of heart failure	1
Hypertension	Systolic hypertension >160mmHg or taking antihypertensive or history of hypertension	1
Age	Age≥75 years	2
Diabetes mellitus	Fasting glucose >125 mg/dL (7 mmol/L) or treatment with an oral hypoglycaemic agent and/or insulin	1
Stroke	history of stroke or TIA or systemic thromboembolism	2
Vascular disease	Prior myocardial infarction or peripheral arterial disease or complex aortic atheroma or plaque on imaging (if performed)	1
Age	Age: 65-74 years	1
: Sex category	Female	
**Risk classification:** 0 low risk; 1 intermediate; ≥2 high risk		
2. **ORBIT**		
Older age	Age≥75 years	1
Anaemia/reduced haemoglobin	Male: Hb<13g/dl or Hct <40%<br>Female: Hb<12g/dl or Hct <36%	2
Bleeding	Any history of GI bleeding, intracranial bleeding, or haemorrhagic stroke	2
Insufficiency of renal function	eGFR<60ml/min	1
Treatment with antiplatelet	Aspirin or thienopyridines	1

Risk classification: 0-2 low risk; 3 intermediate risk; 4-7 high risk

Table S3: Cox proportional hazard of discontinuation at different days gap

Comparisons	Main analysis	Sensitivity analyses	
	60 days gap	30 days gap	90 days gap
	Hazard ratio (95% CI)	Hazard ratio (95% CI)	Hazard ratio (95% CI)
Warfarin vs. apixaban	1.78 (1.54-2.06)	1.84 (1.58-2.15)	1.49 (1.27-1.74)
Warfarin vs. Dabigatran	1.56 (1.29-1.87)	1.70 (1.39-2.08)	1.35 (1.11-1.64)
Warfarin vs. rivaroxaban	1.52 (1.33-1.73)	1.50 (1.30-1.72)	1.28 (1.11-1.47)
Dabigatran vs. apixaban	1.14 (0.94-1.39)	1.08 (0.88-1.33)	1.10 (0.90-1.35)
Dabigatran vs. rivaroxaban	0.98 (0.81-1.17)	0.88 (0.72-1.07)	0.94 (0.78-1.15)
Rivaroxaban vs. apixaban	1.17 (1.03-1.34)	1.23 (1.07-1.41)	1.17 (1.02-1.34)

Table S4: Predictors of OAC discontinuation at 30-days of discontinuation gap

Variables	Multivariable analysis	
	OR (95% CI)	p-value
**Age category**		
<65 years	Reference	
65-74 years	0.62 (0.53-0.72)	<0.001
≥75 years	0.68 (0.60-0.79)	<0.001
**Rurality**		
Major cities	1.00 (0.64-1.59)	0.99
Inner regional	0.92 (0.60-1.47)	0.73
Outer regional	1.26 (0.82-2.00)	0.31
Remote/very remote	Reference	
**Comorbidities**		
Hypertension	0.76 (0.68-0.85)	<0.001
Diabetes mellitus	0.90 (0.80-1.01)	0.078
Vascular disease	1.10 (0.99-1.22)	0.089
Arthritis	0.83(0.75-0.92)	<0.001
**OAC**		
Dabigatran	Reference	
Warfarin	1.73 (1.44-2.10)	<0.001
Rivaroxaban	1.12 (0.93-1.35)	0.26
Apixaban	0.91 (0.75-1.11)	0.34

Table S5: Predictors of OAC discontinuation at 90-days of discontinuation gap

Variables	Multivariable analysis	
	OR (95% CI)	p-value
**Age category**		
<65 years	Reference	
65-74 years	0.60 (0.51-0.71)	<0.001
≥75 years	0.67 (0.57-0.78)	<0.001
**Rurality**		
Major cities	1.21 (0.71-2.18)	0.50
Inner regional	1.11 (0.66-2.00)	0.71
Outer regional	1.59 (0.94-2.85)	0.10
Remote/very remote	Reference	
**Comorbidities**		
Congestive heart failure	0.87 (0.76-0.99)	0.040
Hypertension	0.73 (0.65-0.83)	<0.001
Diabetes mellitus	0.84 (0.73-0.96)	0.013
Stroke	0.92 (0.86-0.99)	0.037
Arthritis	0.78 (0.70 -0.89)	<0.001
**OAC**		
Dabigatran	Ref	Ref
Warfarin	1.45 (1.17-1.80)	<0.001
Rivaroxaban	1.10 (0.90-1.37)	0.35
Apixaban	0.94 (0.76-1.17)	0.56

References

23. Bezabhe WM, Bereznicki LR, Radford J, et al. Oral Anticoagulant Treatment and the Risk of Dementia in Patients With Atrial Fibrillation: A Population-Based Cohort Study. J Am Heart Assoc. 2022;11(7):e023098.

24. Lip GY, Nieuwlaat R, Pisters R, et al. Refining clinical risk stratification for predicting stroke and thromboembolism in atrial fibrillation using a novel risk factor-based approach: the euro heart survey on atrial fibrillation. CHEST. 2010;137(2):263-72.

25. O'Brien EC, Simon DN, Thomas LE, et al. The ORBIT bleeding score: a simple bedside score to assess bleeding risk in atrial fibrillation. Eur Heart J. 2015;36(46):3258-64.
